# Polypoidal Intestinal Metaplasia and Dysplasia of the External Urethral Meatus

**DOI:** 10.1155/2012/703908

**Published:** 2012-12-24

**Authors:** Mary Mathew, Bhavna Nayal, Lakshmi Rao, Raghunath Narayanan Unni, Joseph Thomas

**Affiliations:** ^1^Department of Pathology, Kasturba Medical College, Manipal University, Manipal 576104, India; ^2^Department of Urology, Kasturba Medical College, Manipal University, Manipal 576104, India

## Abstract

Urethral mucosa with intestinal metaplasia and dysplasia is a rare occurrence. To date only a single case has been reported in a male with long-standing urethral stricture. We present a 33-year-old female with polypoid intestinal metaplasia and dysplasia of the external urethral meatus in the absence of an inciting factor. Intestinal metaplasia of the urethral mucosa may undergo dysplasia, emphasizing the necessity of a high degree of clinical suspicion and vigilant pathological examination of these lesions.

## 1. Introduction

Intestinal metaplasia of the urethral mucosa is extremely rare with very few cases documented in females [1–4] and is still rarer in males [5, 6]. We present a 33-year-old female patient with polypoid intestinal metaplasia with dysplasia of the external urethral meatus, presenting clinically as urethral caruncle. To the best of our knowledge, this is the first case of dysplasia arising in a polypoid intestinal metaplasia to be reported in a female patient in English literature.

## 2. Case Report

**Figure 1:**
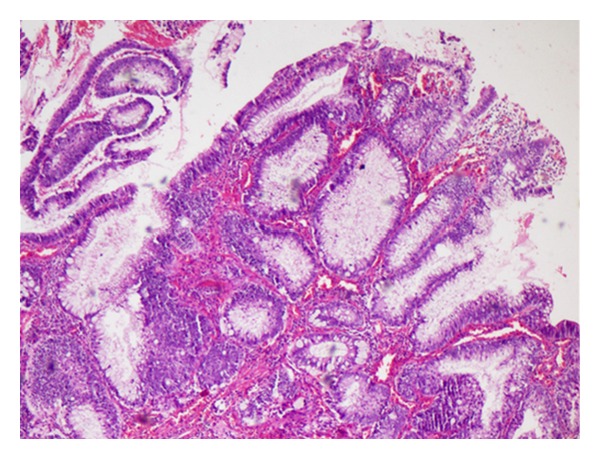
Ulcerated polypoidal epithelium showing extensive intestinal metaplasia and dysplasia (H&E, 10x).

**Figure 2:**
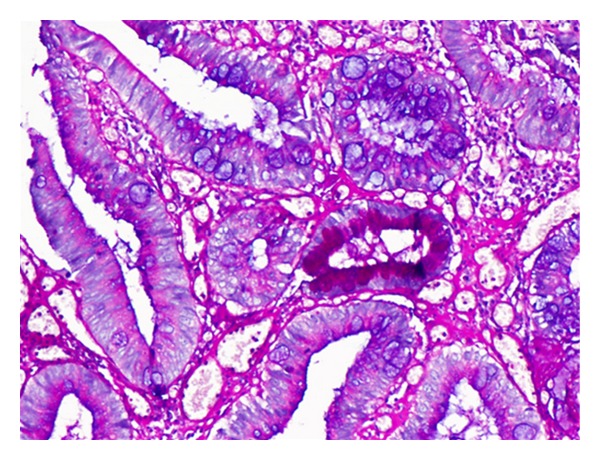
Metaplastic epithelium showing glands lined by goblet cells (periodic acid Schiff with Alcian blue, 20x).

A 33-year-old female patient presented to the Urology clinic with complaints of poor stream of urine and bleeding per urethra since 8 days. There was no history of fever, hematuria, or dysuria. On examination, a polypoid mass measuring 1 × 1 cm was seen in the external urethral meatus. Routine urine microscopy, hematological and biochemical investigations were normal. No abnormalities were detected in ultrasound abdomen. A clinical diagnosis of urethral caruncle was proffered. The mass was excised and sent for histopathological examination. Microscopy revealed an ulcerated polypoidal lesion lined by squamous epithelium with extensive intestinal metaplasia and dysplasia (Figure 1). The metaplastic intestinal glands were lined by columnar cells and goblet cells which were demonstrated by Periodic acid Schiff with Alcian blue (Figure 2). The final diagnosis of polypoidal intestinal metaplasia with dysplasia was rendered. The patient is currently asymptomatic after 1 month of follow-up.

## 3. Discussion

The mucosa of the urinary tract, commonly the urinary bladder and occasionally the ureter and renal pelvis, may undergo intestinal metaplasia secondary to chronic infections, schistosomiasis, calculi, or exstrophy. Intestinal metaplasia of the urethra is extremely rare. Columnar epithelial metaplasia associated with goblet cells alone is referred to as incomplete intestinal metaplasia. The presence of goblet cells along with Paneth cells and argentaffin cells is considered as complete intestinal metaplasia [5, 6]. The present case demonstrated incomplete intestinal metaplasia and no Paneth cells were noted.

The presence of intestinal mucosa in the urethra is postulated to have an embryologic basis. The urethra develops from the urogenital sinus which lies anterior to the urorectal septum. The posterior part forms the rectum. It has been hypothesized that the source of the intestinal epithelium in the urinary tract could be due to the sequestration of aberrant cloacogenic colonic glands in the urethra during the embryologic development of the cloaca [1, 6].

The metaplastic epithelium of the urethra may also undergo dysplasia. A single case of prostatic urethral intestinal metaplasia with dysplasia secondary to long standing stricture of the membranous and bulbous urethra has been documented [5].

 Glandular metaplasia and dysplasia are both premalignant conditions and may give rise to urethral adenocarcinomas or may be adjacent to an invasive malignancy [1, 7]. The present case showed dysplasia of the metaplastic intestinal epithelium. However, no adjoining invasive neoplasm was identified in the urinary tract.

Polypoid or local urethral lesions are treated by transurethral resection. An annual surveillance by cysto-urethroscopy and biopsy is recommended for detection of any neoplastic process [8].

## 4. Conclusion

Intestinal metaplasia in the urethral mucosa is a rare lesion which may undergo dysplasia and regular follow-up is required to detect malignant transformation. Heightened awareness of this lesion, a high degree of clinical suspicion, and careful pathological examination are imperative for early detection. The presence of both intestinal metaplasia and dysplasia is a unique feature in this case.
